# Maternal and child health service delivery in conflict-affected settings: a case study example from Upper Nile and Unity states, South Sudan

**DOI:** 10.1186/s13031-020-00272-2

**Published:** 2020-05-27

**Authors:** Samira Sami, Augustino Mayai, Grace Sheehy, Nicole Lightman, Ties Boerma, Hannah Wild, Hannah Tappis, Wilfred Ochan, James Wanyama, Paul Spiegel

**Affiliations:** 1 Center for Humanitarian Health, Department of International Health, Johns Hopkins Bloomberg School of Public Health, Baltimore, MD USA; 2 University of Juba, and The Sudd Institute, Juba, South Sudan; 3 Department of Population, Family and Reproductive Health, Johns Hopkins Bloomberg School of Public Health, Baltimore, MD USA; 4 Department of International Health, Johns Hopkins Bloomberg School of Public Health, Baltimore, MD USA; 5 Centre for Global Public Health, University of Manitoba, and Countdown to 2030 for Women’s, Children’s and Adolescents’ Health, Winnipeg, Canada; 6 Stanford University School of Medicine, 291 Campus Drive, Stanford, CA 94305 USA; 7 United Nations Population Fund South Sudan, Juba, South Sudan

**Keywords:** South Sudan, Conflict, Health system, Displaced populations, Maternal health, Child health

## Abstract

**Background:**

Decades of war left the Republic of South Sudan with a fragile health system that has remained deprived of resources since the country’s independence. We describe the coverage of interventions for women’s and children’s health in Upper Nile and Unity states, and explore factors that affected service provision during a protracted conflict.

**Methods:**

We conducted a case study using a desk review of publicly available literature since 2013 and a secondary analysis of intervention coverage and conflict-related events from 2010 to 2017. During June through September 2018, we conducted 26 qualitative interviews with technical leads and 9 focus groups among health workers working in women and children’s health in Juba, Malakal, and Bentiu.

**Results:**

Coverage for antenatal care, institutional delivery, and childhood vaccines were low prior to the escalation of conflict in 2013, and the limited data indicate that coverage remained low through 2017. Key factors that determined the delivery of services for women and children in our study sites were government leadership, coordination of development and humanitarian efforts, and human resource capacity. Participants felt that national and local health officials had a limited role in the delivery of services, and financial tracking data showed that funding stagnated or declined for humanitarian health and development programming during 2013–2014. Although health services were concentrated in camp settings, the availability of healthcare providers was negatively impacted by the protracted nature of the conflict and insecurity in the region.

**Conclusions:**

Health care for women and children should be prioritized during acute and protracted periods of conflict by strengthening surveillance systems, coordinating short and long term activities among humanitarian and development organizations, and building the capacity of national and local government officials to ensure sustainability.

## Background

Decades of civil war with the Sudanese government left the Republic of South Sudan with a fragile health system that has remained deprived of resources since the country’s independence in 2011. The world’s youngest nation continued to struggle following a resurgence of complex politically- and ethnically-charged civil conflict in 2013. The recent conflict initially resulted from a political struggle between the President, a member of the majority ethnic group, the Dinka, and Vice President, a member of the second largest ethnic group, the Nuer. In December 2013, fighting between Dinka and Nuer members of the presidential guard began in the capital city of Juba and rapidly spread throughout the country, ravaging the Greater Upper Nile and Unity regions [1, 2]. Fighting further intensified with the fragmentation of political groups that exploited longstanding intercommunal tensions that are often incited by cattle raiding [3]. The violence forced many people to flee their homes as refugees and internally displaced persons (IDPs), with some of the IDPs seeking shelter in the United Nations’ (UN) protection of civilians (POC) sites located in Juba (Central Equatoria state), Malakal (Upper Nile state), and Bentiu (Unity state) [3]. Indiscriminate killings of civilians, including attacks against health facilities, caused humanitarian agencies to abandon these areas and evacuate non-local staff.

Fighting in Upper Nile and Unity continued well into 2015, with minor conflicts continuing despite a peace agreement brokered in August 2015. During this time, militia purposefully destroyed stocks of medicine and supplies in the states’ towns, and prevented critical humanitarian assistance from reaching the POC sites [4, 5]. When health workers were abducted or killed, humanitarian agencies were frequently forced to suspend operations, returning to the field when the situation permitted. Following intense fighting in July of 2016, displacement reached unprecedented levels. By the end of 2017, more than 1.9 million persons became internally displaced and over 2 million fled as refugees to neighboring countries [6]. Most of these individuals were displaced multiple times. During this period, a maternity ward operated by an international non-governmental organization (INGO) in the Juba POC site was shelled [7]. By 2017, widespread conflict caused deteriorating security even in those states that had benefitted from relative stability and peace. The terms of the Agreement for the Resolution of Conflict in South Sudan had been repeatedly violated by both sides. As officials met to revitalize previous peace agreements, a man-made famine was declared in Unity state after relentless war and drought [8].

The humanitarian crisis in South Sudan, exacerbated by a collapsed health system that was weak prior to the conflict, poses a challenge to protecting and improving the health of women and children. Although the South Sudan Ministry of Health (MOH) drafts policies and strategic plans to guide reproductive, maternal, newborn, child, and adolescent health (RMNCAH) services, most of the health system is run by externally funded INGOs [9]. Despite the efforts of humanitarian organizations, protracted conflict has depleted a skilled health workforce capable of implementing these services and only an estimated 25 to 30% of the South Sudanese population had access to health care in 2015 [10]. The high level of maternal mortality (789 per 100,000 live births) and under-five mortality (96.4 per 1000 live births) in South Sudan are undoubtedly a result of these factors [11, 12]. Few studies describe the factors that influence the effectiveness of health interventions for women and children in conflict-affected countries, including South Sudan.

This study is part of a multi-country study coordinated by the BRANCH (Bridging Research & Action in Conflict Settings for the Health of Women & Children) [13] Consortium focused on reproductive, maternal, newborn, child and adolescent health and nutrition research in ten conflict-affected countries: Afghanistan, Colombia, DRC, Mali, Nigeria, Pakistan, Somalia, South Sudan, Syria and Yemen. In this paper, we report findings from the case study in South Sudan. We examine maternal and child health service delivery in a protracted humanitarian crisis during the period of intermittent acute conflict spanning from 2013 to 2017 in two heavily conflict-affected states, Upper Nile and Unity. We aim to: 1) describe the coverage of RMNCAH interventions, and 2) explore the factors that influence the provision of RMNCAH services in this context.

## Methods

### Design and setting

We used a case study design, modified from a standardized protocol agreed upon by the BRANCH Consortium according to context, to document the delivery of health interventions for women and children in South Sudan during December 2013 to 2017. Secondary data were obtained from national population-based surveys, health information systems, and a desk review of published and unpublished documents. We also collected primary data using qualitative methods in two conflict-affected regions of South Sudan, Upper Nile and Unity states. These sites were selected due to high estimates of mortality and displacement during the study period. Service delivery at these sites included camp and non-camp settings, as well as government and non-government facilities. The number of IDPs peaked in mid-2015 [12]. Refugees, primarily from Sudan, were hosted in camps in Upper Nile and Unity, with a population of 145,985 and 121,855, respectively [14]. By the end of 2017, there were an estimated 219,645 IDPs in Upper Nile (with 24,424 of IDPs living in Malakal POC site) and 540,085 IDPs in Unity (with 112,140 living in Bentiu POC site) [12, 15].

### Data collection

Multiple data sources were synthesized in this case study to assess health service delivery during the 4-year period of intense conflict that began in December 2013. Study co-investigators from Johns Hopkins Bloomberg School of Public Health (JHSPH) and the University of Juba collaborated on study design and oversight. A team of research assistants with experience in public health from the University of Juba led primary data collection activities in South Sudan. A 3-day training, facilitated by two co-investigators, was held in Juba with the local research team to orient them to the study objectives, methodology, instruments, and research ethics, and to conduct a pilot test of the study instruments. Ethical approval for this case study was received from the Republic of South Sudan’s Ministry of Health. JHSPH determined that this study was non-human subjects research and therefore exempting the study from institutional review board oversight.

Starting with a desk review of peer-reviewed literature on health services in South Sudan, the investigators searched for articles from 2013 to present using the PubMed database. The search term “South Sudan” was combined with MESH terms and general search terms related to the delivery of RMNCAH interventions. Our strategy yielded 107 results, 28 of which were relevant to the study question. Articles that did not address the timeframe and health interventions of interest were excluded from our review. We also searched the grey literature for program reports related to the health system in South Sudan using humanitarian websites such as Relief Web, Humanitarian Response, and World Health Organization (WHO) Health Cluster. From this search, we obtained an additional 38 reports that were compiled and managed for data extraction using Microsoft Excel. Documents gathered from the desk review were used to provide additional context on health system barriers in South Sudan.

Secondly, we consulted with stakeholders in the MOH, UN agencies and INGOs to access data from 2010 to 2017 on population estimates, intervention coverage, and conflict-related events including fatalities. Four national population-based surveys were conducted in the past 7 years: 2010 Multiple Indicator Cluster Survey (MICS) [16], 2012 Expanded Programme on Immunization (EPI) coverage surveys [17], and 2011 and 2015 Lot Quality Assurance Sampling (LQAS) surveys [18]. However, due to insecurity during data collection, the 2012 EPI survey did not include Unity or Upper Nile states and the 2015 LQAS survey did not include Unity state. Facility-derived data describing select RMNCAH service indicators were also obtained from the Health Management Information System (HMIS) reports from 2010 to 2017. We compiled these surveys and extracted data on five RMNCAH coverage indicators: antenatal care- at least one visit (ANC1); antenatal care- at least four visits (ANC4); institutional delivery; diphtheria, tetanus and pertussis vaccine (DPT3); and measles vaccine. Indicators were selected based on consistency across data sources. The Armed Conflict Location & Event Data (ACLED) provided estimates of violence-related events and fatalities from 2013 to 2017, while the population figures from South Sudan’s National Bureau of Statistics were used to determine intervention coverage and violence-related death rates [19].

Lastly, we used qualitative methods to collect primary data in Juba, Malakal, and Bentiu. We conducted data collection during the period from June to September 2018. We compiled a list of 23 agencies that have a role in women and children’s health care such as donor institutions, government offices within the Ministry of Health, and humanitarian health organizations, and purposively sampled participants from these organizations. All technical leads who worked in these agencies at the national level (Juba) and in the field (Bentiu and Malakal) and had 30 or more days of experience working in South Sudan were invited to participate in an in-depth interview. Twenty-six participants were interviewed by a trained researcher from the University of Juba (see Table 1). Interviews were conducted in person, lasted an average of 40 min, and were audio recorded after receiving the participant’s consent. A semi-structured interview guide, developed by study co-investigators, gathered detailed information on the impact of insecurity, decision-making processes, and other factors regarding RMNCAH service delivery.

**Table 1:** Participant characteristics for qualitative data, June – September 2018

Variable	Focus groups (***N*** = 9)		In-depth interviews (***N*** = 26)	
	No.	(%)	No.	(%)
**Sample size**				
Number of participants	58		26	
**Location**				
Juba, Central Equatoria	0	(0.0)	7	(26.9)
Malakal, Upper Nile	33	(56.7)	9	(34.6)
Bentiu, Unity	25	(43.1)	10	(38.4)
**Type of staff**				
Community health worker	34	(58.6)	0	(0.0)
Facility health worker	24	(41.4)	0	(0.0)
Technical lead	0	(0.0)	23	(88.5)
Government official	0	(0.0)	3	(11.5)
**Sex**				
Male	32	(55.2)		
Female	26	(44.8)		
**Mean age, years**	34.6 ± 8.6			
**Education completed**				
None	2	(3.4)		
Primary	22	(37.9)		
Secondary or higher	34	(58.6)		
**Mean time employed by agency, years**	2.2 ± 1.9			

A team of researchers from the University of Juba traveled to Bentiu and Malakal to conduct focus group discussions with facility and community-based health workers who provided health services to displaced persons during conflict. Co-investigators developed a discussion guide to understand how cultural factors, insecurity, displacement, and disease outbreaks influenced health service provision for women and children. All available health workers residing in the POC sites were invited to participate. Five focus groups were held with health workers in Upper Nile and four focus groups were conducted in Unity, representing a total of 58 health workers (Table 1). Group discussions were held separately for community and facility health workers; 41% of the total participants were facility-based health workers and 59% were community health workers. The average age of participants was 35 years and the average number of years employed by their agency was 2. Focus group discussions comprised five to eight participants, took approximately 90 min, were conducted in Arabic and English, and audio recorded with participant consent.

### Analysis

For the document analysis, an annotated bibliography was developed using Microsoft Excel. Compiled materials were reviewed for information about factors within the health system that influenced health service delivery. Relevant information was summarized into a Microsoft Word document by the study team. Data extracted on the five RMNCAH coverage indicators were synthesized to describe patterns and trends in coverage estimates over time for South Sudan and Unity and Upper Nile regions. ACLED data were summarized to present number of violent events and fatalities that occurred from 2010 to 2017. For the qualitative data, recordings were translated into English and transcribed verbatim into a Word document by the local research team. English recordings were transcribed by a US-based transcription company. Researchers at the University of Juba and JHSPH developed an initial codebook using inductive and deductive approaches (see Table 2). Transcripts and field notes were then uploaded into NVivo (QSR International, Version 11.4.0) for further analyses using the final codebook. A research assistant from JHSPH coded the transcripts using NVivo, and drafted analytical memos that were reviewed by study co-investigators for additional insight. Through the thorough review of transcripts and memos, we identified emergent patterns, themes and subthemes.

**Table 2:** Summary of emergent themes from qualitative data

Theme	Emergent Sub-Theme
Security context	*Impact:* Impact of the changing security context on health services and decision-making
	*Coping:* How humanitarian agencies cope in the current environment to continue health services
Displaced populations	How the influx of displaced populations influenced health services including receipt of additional support in response to an influx
Competing priorities	How competing priorities impact RMNCAH service delivery (e.g. time or resources) and how these competing priorities are managed
	*Disease outbreak:* How disease outbreaks have affected delivery of RMNCAH services including receipt of additional support to address the outbreak
Leadership and governance	*Decision-making:* Factors that inform which health interventions to deliver including financial costs and expertise of staff
	*Coordination:* How agencies coordinate with other stakeholders
	*Restrictions*: Requirements that agencies must adhere to for working in the area (e.g. special permissions)
	*Governance:* Policies, guidelines, or regulations that determine or affect health interventions or service provision
Health workforce	*Impact*: How the availability of health workers has affected RMNCAH services
	*Turnover and recruitment:* Management of health workforce and challenges faced in recruitment of health workers
Information and research	*Evidence*: Sources of information that were used to inform RMNCAH interventions
Service delivery	*Supply shortages:* Availability of medical supplies for RMNCAH services and adjustments made to services because of shortages
	*Access:* Description of populations that were hard to access and efforts made to reach these populations
Culture	*Acceptability:* How cultural beliefs have influenced the acceptability or use of RMNCAH interventions
	*Service provision:* How cultural beliefs have influenced service provision or how agencies have worked to address cultural barriers that have impacted use of RMNCAH services

## Results

**Fig. 1:**
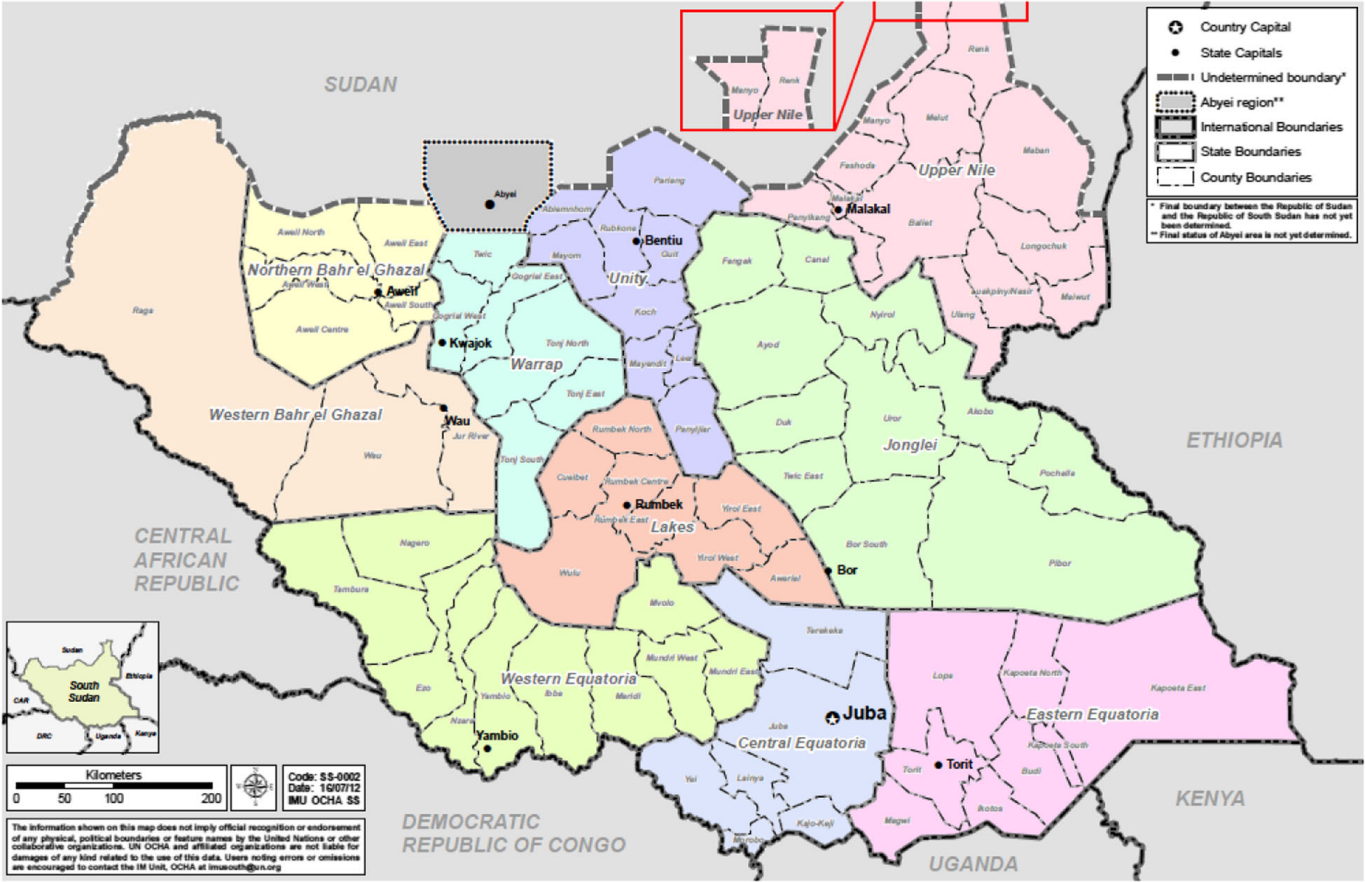
Map of South Sudan and Upper Nile and Unity States Source: UN Office for the Coordination of Humanitarian Affairs, 2012

**Fig. 2:**
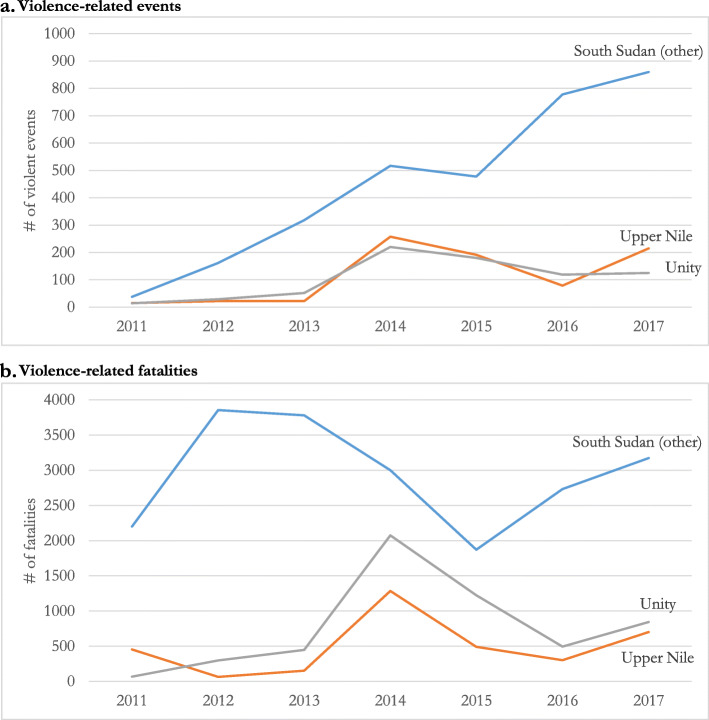
Average number of violence-related events and fatalities in South Sudan, 2011–2017 a. Violence-related events b. Violence-related fatalities

The number of violence-related events and fatalities increased rapidly from 2013 to 2014 in Upper Nile and Unity states, reaching a peak of more than 3360 violence-related fatalities and 478 violence-related events in 2014 (Figs. 1 and 2) [19]. Although the number of violence-related fatalities decreased from 2015 to 2016 in both states, the number of fatalities rose in 2017 following renewed insecurity in Upper Nile and Unity states [19]. Armed conflict in Unity and Upper Nile was also an important driver of the increase in violence-related events and fatalities. Compared to the national annual rate of violence-related deaths at 0.7 per 1000 persons, Unity and Upper Nile experienced a higher annual death rate of 3.8 per 1000 and 1.1 per 1000, respectively [19].

### Availability of health services for women and children during conflict

**Fig. 3:**
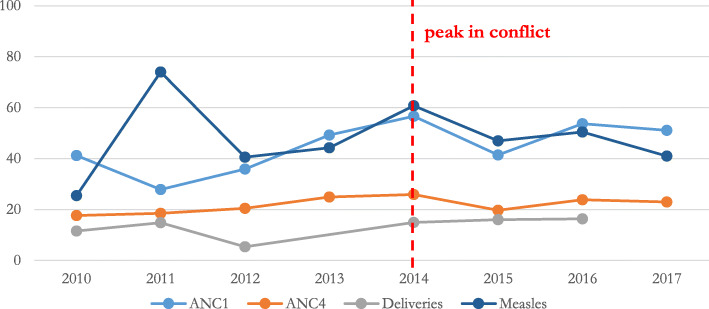
Health facility data-derived intervention coverage estimates for South Sudan, 2010–2017

While the results are not directly comparable, we present coverage rates reported in four national surveys from 2010 to 2015 (see Table 3). Coverage was very low for all health interventions prior to the 2013 conflict: ANC1 (2010: 40.3%, 2011: 44.6%), ANC4 (2010: 17.3%, 2011: 20.0%), institutional delivery (2010: 11.5%, 2011: 16.2%), DPT3 vaccine (2010: 15.1%, 2011: 19.5%), and measles vaccine (2010: 26.3%, 2011: 38.0%). There was little difference between Upper Nile and Unity for ANC1, ANC4, and institutional deliveries in the MICS 2010 or the LQAS 2011. Compared to the national average, Unity had lower coverage for DPT3 and measles vaccinations in 2011. During 2010–2015 the gap in coverage increased between Upper Nile and national estimates as most of the reported indicators for Upper Nile remained nearly the same. Health facility-derived data at the national level followed similar trends as population-based survey data for ANC1, ANC4, and measles, with low coverage rates and little improvement over time. However, data from health facilities indicated institutional delivery rates were lower than population-based survey results in the most recent survey (see Fig. 3).

**Table 3:** Population-based survey data intervention coverage estimates, 2010–2015

Indicator	2010^**a**^	2011^**b**^	2012^**c**^	2015^**d**^
**Antenatal care: at least 1 visit**				
South Sudan	40.3%	44.6%	–	69.0%
Upper Nile	37.8%	–	–	63.1%
Unity	31.5%	–	–	–
**Antenatal care: at least 4 visits**				
South Sudan	17.3%	20.0%	–	22.6%
Upper Nile	19.8%	17.4%	–	22.4%
Unity	12.1%	11.2%	–	–
**Institutional delivery**				
South Sudan	11.5%	16.2%	–	27.2%
Upper Nile	8.1%	16.7%	–	13.7%
Unity	11.4%	16.9%	–	–
**DPT3 vaccine**				
South Sudan	15.1%	19.5%	46.0%	33.8%
Upper Nile	14.9%	22.5%	–	24.6%
Unity	9.7%	10.5%	–	–
**Measles vaccine**				
South Sudan	26.3%	38.0%	46.0%	48.9%
Upper Nile	32.8%	42.4%	–	45.6%
Unity	19.8%	34.9%	–	–

^a^ MICS 2010

^b^ LQAS 2011

^c^ EPI 2012

^d^ LQAS 2015

### Factors influencing RMNCAH service delivery in upper Nile and Unity states

#### International factors

Interviews with technical leads revealed that several factors related to international policies and practices influenced the types of health services that were delivered during conflict. The availability of funds was commonly described as a major factor that influenced decision-making. Although the total amount of humanitarian aid increased by 107% from 2013 (USD 771.9 million) to 2014 (USD 1.6 billion), the funding allocated for humanitarian health increased by 4% (USD 70.8 thousand to USD 73.5 thousand) [20]. In 2014, during the peak of the conflict, development aid for health decreased by 60% from 2013 (USD 72.5 thousand to USD 29.4 thousand) [21]. Participants stated that access to long-term funding beyond one or 2 years became scarce, and grants were offered in 3-month increments due to donor concerns about insecurity and transparency. Interview participants described an environment of limited funding for health service delivery with pervasive “*donor fatigue*” due to the recurring emergencies. Specifically, for family planning services, a UN participant overseeing health programs in South Sudan stated, “*The funding in terms of reproductive health has dramatically reduced within the last 2 years. We used to support many partners and we are really not covering all areas because we don’t have enough funding*.” Infrastructure was also a pressing concern among participants: “*You can’t be able to provide quality maternal services for mothers without infrastructure, without an operating theater. The investment in infrastructure is weak, both because the government does not have a lot of money, coupled with the fact that most donors that support this sector are reluctant to invest in infrastructure because of the destruction of facilities*.” (Health worker, Unity).

#### National factors

At the national level, the government health budget was affected considerably by the depreciation of the local currency. Another UN technical lead based in Juba stated, “*Since 2013, you will see the government portion of the [health] budget has only shrunk. That means in terms of health service provision, there are huge gaps that are supposed to be covered by humanitarian aid.*” In 2013, 2% of total government spending in fiscal year 2013/2014 was spent on health compared to 7% in fiscal year 2012/2013 [22]. The two main development funding mechanisms in South Sudan, Health Pooled Fund and World Bank, support health services and emergency operations that are expected to reduce maternal and child mortality in Upper Nile and Unity since 2012 [23]. Meanwhile, humanitarian agencies are also conducting emergency response in these regions through the humanitarian cluster system. The dynamic context within the country combined with hyperinflation made costing interventions challenging: “*The current cost cannot be equated to the previous cost because the situation has changed and the context has absolutely changed. The amount that was allocated for transportation is not the same as the amount allocated now because everything has gone up*.” (NGO technical lead, Juba). While the MOH oversaw the development of policies and guidelines, including the 2013–2016 Reproductive Health Strategic Plan, over 80% of health services were delivered by INGOs [9].

Government facilities were described as extremely under-resourced and understaffed, as health workers were displaced, killed, or moved to better-paying opportunities. Participants repeatedly stated that the lack of government investment in the health workforce led to unequal geographical distribution among recently graduated midwives and nurses, with few traveling to work in government facilities in Upper Nile and Unity. The protracted nature of the conflict has contributed to high turnover of staff and continued to negatively impact the availability of midwives in these regions. These issues continue to be an important barrier for safe maternal and newborn care.

#### Insecurity

Ongoing insecurity in South Sudan impeded the ability of the health system to provide timely and consistent services for women and children. At least 29 health facilities were looted or destroyed between December 2013 and 2016, and 24 humanitarian workers were killed in 2016 alone [7]. Since 2013, most health services in Unity and Upper Nile states were concentrated in POC sites. Even as conflict diminished at the national level, health service delivery varied considerably across the country. In Upper Nile, focus group participants spoke about expanding services outside of the Malakal POC site, including through mobile clinics. However, participants described insecurity as a challenge for providing services outside of POC sites. They estimated that only 60% of health facilities in Upper Nile were functional in 2017. Health workers in Unity also described ongoing tensions along ethnic lines, limiting which parts of the region they could safely access: “*Sometimes if they know my brother is on the other side of the conflict, they will not allow me in. They do not think that this is a health worker who is not involved in politics. It happened in January when we arrived in a vehicle during the vaccine campaign. They told us that we will not even vaccinate any single child there. They will kill us and the vaccines would remain with them. This is a thing that affects service delivery to the community*.” Although POC sites were generally considered safer than non-camp settings for the population in general, security threats within the POC sites remained, including individual attacks, robberies, and overcrowding. Many camps were considered dangerous and unprotected at night: “*Health workers cannot come for night shift because the route from home to the facility is blocked with people fighting. Even the UN does not come here. This has already affected service delivery, because if she [a health worker] is supposed to deliver a mother that mother will not get services that night*.” (Health worker, Unity). The availability of medical equipment and supplies also faced significant disruptions during the conflict, leading to stock outs of essential medicines for RMNCAH services. Poor road conditions and attacks against humanitarian workers made transportation of supplies by land challenging and by air extremely costly.

Participants widely agreed that women and children were most affected by conflict and they have suffered the greatest health impacts: “*From my observation, the most affected groups are women and children. In most cases, when there are security issues, you realize that in these locations, you almost always find women and children. They are the ones that are left behind without services*.” (Health worker, Unity). Many participants considered maternal health care to be one of the services most severely affected by the insecurity, contributing to the high maternal mortality rate in the country. Subsequently, women have been forced to give birth at home or risk their lives seeking delivery care in a facility: “*It’s difficult for the women because some of them are still in the village during this conflict. There’s no health facility outside the POC in the village. They must come inside the POC for them to get help or treatment. Because there’s no health facility, they just deliver at home. When someone is having complication or bleeding, it’s difficult for them to walk and it takes 2 or 3 days of carrying that patient to Bentiu POC because there’s no transport or ambulance that can bring them.”* (Health worker, Upper Nile). During intense periods of conflict, routine pediatric care was also disrupted, including the provision of vaccination, which made children vulnerable to outbreaks such as cholera, meningitis, and measles. Health workers described encountering children who had never received health care and were presenting alone at the health facility because they had been orphaned due to the conflict. Participants who worked in the POC sites described a mental health crisis among youth who were traumatized by the war, leading many to consider suicide.

#### Organizational and coordination level

At the organizational and coordination level, decisions regarding what RMNCAH services to prioritize were largely based on the capacity of frontline health workers to administer services. The lack of trained medical personnel was described as a significant barrier for delivering quality maternal health services. Midwives and doctors were lacking, while limited training to build the capacity of lay health workers exacerbated shortages in skilled delivery and immediate postnatal care. During periods of intense conflict, community health workers were often described as the only available service providers for maternal and child health: “*The advantage we [health organization] have is that we have a very good number of locally recruited staff. So many times, the other [non-local] staff may be affected, but the ones who were locally recruited are adjusting faster than the rest. So it helps, it helps a lot*.” (NGO technical lead, Unity).

Organizations described coordination as an important factor that influenced RMNCAH services. The UN cluster approach was used to coordinate health delivery services, but this approach was largely reliant on the capacity of organizations and their access to populations. At the national level, organizations perceived coordination to be weak in the health cluster and Health Pooled Fund due to the government’s limited technical capacity. However, on the frontlines, UN agencies and NGOs felt coordination was successful among technical working groups; thus, facilitating efforts to streamline and collaborate on assessments that were later used to inform decision-making: “*Just last year [2017], an assessment was done in Malakal town. It was inter-cluster and formed the basis of us knowing what areas in town to go to and where to start providing services*.” Data were often scarce and quickly became out-of-date due to the dynamic context, but rapid assessments along with routine data and expert knowledge informed the prioritization of RMNCAH services during periods of conflict. Study participants did not make substantial reference to international standards, key guidelines, and peer-reviewed articles when asked about decision-making processes for key RMNCAH interventions. Since data are mostly scarce and not readily available, most participants draw upon needs assessment findings and the government’s basic package of health and nutrition services to inform programming.

#### Community level

Cultural beliefs and practices at the community level, in addition to literacy and gender inequality, were key factors considered to shape women and children’s use of health services. Vaccines and treatment of childhood illnesses were the most negatively affected by cultural and religious beliefs leading to heavy reliance on traditional medicines and providers. Health workers in the focus groups described extensive community engagement activities aimed at understanding these concerns in the community. Health education was the main strategy frequently referred to as a means of addressing sociocultural barriers. Participants also described the gendered dynamics of conflict that affected health service delivery. A community health worker in Upper Nile described, “*The burden is placed on the woman to provide food to their children. This is a challenge we are facing here. A mother goes to get fish from the river, then going to sell it in the market to make a profit. This is all done at the expense of breastfeeding the baby.”* Men were described as key decision makers in the utilization of family planning and many were resistant to its use because of negative misconceptions such as “*women become sterile after using contraception*” and “*condoms are only used by sick people.*” Health workers felt women, particularly those living outside of the POC site who travel to the camp for health services, were at risk of rape and violence, which may lead to unwanted pregnancy and sexually transmitted infections.

## Discussion

Improving outcomes for women and children in conflict-affected countries requires consideration of context-specific factors that influence the implementation of life-saving interventions. This study provides insights into RMNCAH service delivery from 2013 to 2017 in two regions of South Sudan heavily affected by conflict. Since the country’s independence following a protracted period of armed conflict, South Sudan has had some of the lowest coverage of RMNCAH indicators in the world. While limited evidence from surveys suggests that some RMNCAH interventions increased coverage from 2010 to 2015 in Unity and Upper Nile, health facility data indicate that coverage of key interventions remained very low in South Sudan.

Although data collection challenges make it difficult to collect reliable data on coverage at the national and state levels, health facility data can provide an insight into trends for key indicators for women and children. To improve the quality and comprehensiveness of coverage estimates based on facility data, further attention must be given to facility-based health information systems. Strengthening surveillance systems is critical for understanding gaps in RMNCAH services, particularly since surveys often exclude insecure regions from their sample. This can be done through reducing the burden on clinical staff, establishing remote support, and providing standardized training opportunities in data collection [6].

Because of the gaps in existing data, organizations that support health service delivery in Upper Nile and Unity frequently conduct rapid assessments based on qualitative interviews to inform their decisions. Linking this information with evidence on high impact, low cost interventions for women and children can strengthen decision-making. For instance, guidelines such as the Inter-Agency Field Manual on Reproductive Health in Humanitarian Settings as well as the Newborn Health in Humanitarian Settings Field Guide describe evidence-based interventions and strategies for dramatically improving maternal and newborn health outcomes [21, 24]. The lack of reference to international standards, guidelines, or peer-reviewed articles among study participants may be due to limited dissemination of resources in country.

Our findings highlight major factors that influenced health service delivery for women and children at the international, national, organizational, and community levels. We identified three main areas according to our qualitative research in Upper Nile and Unity: 1) government leadership; 2) coordination of development and humanitarian efforts; and 3) human resource capacity. First, our study revealed that national and local health officials had a very limited role in the delivery of RMNCAH services for women and children. Although there was minimal donor investment in health due to the lack of strong government capacities, we found few efforts by the international community to build the capacity of the government to lead health service delivery in Upper Nile or Unity. This lack of investment affects the government’s leadership and accountability roles in the future. Although international organizations have invested in the training of local midwives and nurses, the government and donors need to incentivize these new graduates to work in government-run health facilities in the most destitute areas to strengthen the health system in the long term. This can include monetary incentives, housing, and investment in future training opportunities for relocated staff. INGOs will likely continue to be the preferred recipient of development and assistance funds over the government for the foreseeable future because of the lack of confidence of the international donor community in the peace process and lack of institutional capacities. Medium- and long-term strategies need to be created that build the capacity of the MOH at the central, state and county levels with clear benchmarks that build confidence in the government over time. Strong governmental leadership, prioritization of strategic plans, local allocation of funds, and measuring progress to ensure accountability were key activities identified among countries that achieved positive gains in maternal and child health in Africa [25].

Secondly, despite increases in total humanitarian funding from 2013 to 2017 in South Sudan, our study found that donors reduced financial support for development health aid during this period. As mentioned above, humanitarian and development aid is deliberately bypassing the government and provided instead to INGOs. This demonstrates the level of crisis in the country, since development actors have had to transition their services to focus on emergency interventions. Coordination between development and humanitarian actors is an essential way to provide emergency response while setting the stage for longer term development with indicators of measurement and financial reporting. A more coordinated approach among the development and humanitarian actors at the state level will allow for a clearer understanding of roles to avoid duplication of efforts. Successful examples of linking development and humanitarian spheres have been demonstrated in previous crises such as the Philippines, in which preparedness and recovery efforts were funded at the community level to promote health system resilience [26]. To improve longer term presence in Upper Nile and Unity, INGOs can expand services from POC sites to provide health services in other nearby areas as security allows, beginning with community health workers, as well as building the capacity of national NGOs. Dedicating resources to strengthen local capacities for sustainable health services in the country is now more critical as South Sudan implements the Revitalized Agreement for the Resolution of Conflict in South Sudan (R-ARCSS).

Lastly, a major barrier to providing comprehensive RMNCAH services is the availability of a skilled health workforce such as nurses and midwives, particularly outside of Juba. Coupled with poor coverage of health facilities due to destruction and lack of consistent longer term funding to rebuild the infrastructure (which is currently not allowed with the humanitarian or development funds), this likely contributes to high maternal and child mortality in South Sudan. A multitude of factors have contributed to the ongoing severe shortage of human resources in the country. Large turnover among overstretched staff and lack of adjustment of government health worker salaries to account for hyperinflation of the local currency are key issues that cannot be ignored when tackling the health workforce in South Sudan. These challenges can take a generation or more to address; however, task shifting of key lifesaving interventions for women and children can be prioritized in the short term to improve coverage of health services [27]. Administration of misoprostol to prevent postpartum hemorrhage and basic newborn care, including breastfeeding promotion and kangaroo mother care for low birth weight babies, are some examples of interventions that can be introduced or scaled up among lay health workers to improve maternal and newborn care [27, 28].

### Limitations

South Sudan’s National Bureau of Statistics produces annual population estimates that were used as denominators for the intervention coverage estimates. The population figures tend to vary considerably by year, and the uncertainty of these estimates needs to be taken into account. Additionally, health facility-derived estimates of coverage are affected by the data quality and the reporting rates. Poor reporting may indicate no services provided, but in other instances the data did not reach the MOH. We are unable to report state-specific coverage estimates using HMIS data owing partly to lack of reliable population estimates due to the high levels of displacement and the high fluctuations in reporting completeness at the state level, which in some cases may be genuine indications of the inability of the country to provide services and in other cases is simply due to poor reporting. Our results are also limited to the context in Upper Nile and Unity, two areas heavily-affected by conflict, and may not be generalizable to other states in the country. Lastly, data collection for the quantitative data was conducted during 2010–2017 and qualitative interviews and focus groups were conducted in mid-2018 among participants located in South Sudan. The responses of these participants may be subject to recall bias regarding events that occurred in the past.

## Conclusions

South Sudan’s weak health system posed challenges to the health of women and children even prior to the 2013 resurgence of violence after a period of relative peace. This study provides unique insight into coverage of health interventions and factors that affected service provision for women and children during war. During this time, government leadership, human resource capacity, and coordination of development and humanitarian efforts were impeded. Coverage of RMNCAH interventions for women and children in Upper Nile and Unity remains very low, and there is no evidence that national coverage has improved since the conflict peaked in 2014 despite increased humanitarian and development assistance. Coordination between development and humanitarian actors on indicators regarding measurement and financial reporting is an essential way to set the stage for longer term health development in South Sudan. Health care for women and children should be prioritized during acute and protracted periods of conflict. This means task sharing of key lifesaving interventions for women and children can be prioritized in the short term to improve coverage of health services during investments in training institutions and incentives to expand the reach of skilled health workers. Furthermore, efforts are needed to build the capacity of the government to lead health service delivery with closely monitored benchmarks as the context evolves. A medium and longer-term strategy to build the capacity of the government and national NGOs is essential for sustainability and contextualized delivery of RMNCAH services.

## Supplementary information

**Additional file 1.** Interview Guide: Focus Group Discussion Interview Guide: In-Depth Interview with Governing Authorities.

## Data Availability

The datasets used and/or analyzed during the current study available from the corresponding author on reasonable request.

## Abbreviations

ACLED: Armed Conflict Location & Event Data
ANC1: Antenatal care- at least one visit
ANC4: Antenatal care- at least four visits
BRANCH: Bridging Research & Action in Conflict Settings for the Health of Women & Children
DPT3: diphtheria, tetanus and pertussis
EPI: Expanded Programme on Immunization
HMIS: Health Management Information System
IDP: Internally displaced person
INGO: International non-governmental organizations
LQAS: Lot Quality Assurance Sampling
POC: Protection of civilians
MICS: Multiple Indicator Cluster Survey
MOH: Ministry of Health
RMNCAH: Reproductive, maternal, newborn, child, adolescent health
UN: United Nations
WHO: World Health Organization
