# Supplementary material for: Maternal and child health service delivery in conflict-affected settings: a case study example from Upper Nile and Unity states, South Sudan
Source: Confl Health. 2020 May 27;14:34. doi: 10.1186/s13031-020-00272-2 (PMC7254670; doi:10.1186/s13031-020-00272-2)
Supplement: Supplementary file 1 — Additional file 1. Interview Guide: Focus Group Discussion Interview Guide: In-Depth Interview with Governing Authorities. [file 13031_2020_272_MOESM1_ESM.docx]

Interview Guide: Focus Group Discussion

FOCUS GROUP DISCUSSION GUIDE

FRONTLINE HEALTH WORKERS

Topic 1: Introduction

1. We would like to understand what health services you provide for women and children. Can you please describe the services provided at the facility or community level?

Topic 1: Culture

1. We would like to understand what influences the delivery of health interventions for women and children. Can you explain how cultural beliefs have influenced the acceptability of health interventions among community members?

*Example of health interventions*: Family planning, antenatal care, care during childbirth, essential newborn care, postnatal care, PMTCT of HIV, applying nothing to the newborn’s cord, kangaroo mother care, immediate breastfeeding, care for sick children under five, child vaccination, management of acute malnutrition, care for survivors of sexual assault

*Probe*: Can you give me some examples?

1. Does culture affect service provision in one area of health in particular?

*Probe*: Can you give me an example?

1. Can you describe any subpopulations that were particularly hard to access?
2. Can you describe any special efforts to try to access these subpopulations?

Topic 2: Insecurity

1. We understand that working in South Sudan can be difficult given the ongoing insecurity. Can you describe the impact of the changing security context on your work?

*Probe*: Can you give me some examples?

1. How did you manage the security challenges?
2. How was the security situation changed over time?

Topic 3: Population displacement

1. Were there any large influxes of displaced populations? Can you describe how this influx influenced your service provision?

*Probe*: Can you give me some examples?

1. Did you receive additional support to provide services to the influx of population?

Topic 4: Epidemics

1. Were there any epidemics while you were working here? Can you describe how this influenced provision of other services for women and children?

*Probe*: Can you give me some examples?

1. Did you receive additional support to address the epidemic?

Topic 5: Closing Group Discussion

*[Explain that drinks and snacks will be available at the end of the discussion. Summarize the main points from the group discussion and ask for consensus on main points.]*

1. Does anyone have any additional thoughts that can help us understand what is working well in providing health services to women and children?
2. Before we end our discussion today, is there anything else you want to share about challenges you face in your work that were not shared?

Thank you again for your help and for sharing your opinions with us today. We really appreciate your time and contribution. Feel free to ask any questions.

**Interview Guide: In-Depth Interview with Governing Authorities**

IN-DEPTH INTERVIEW GUIDE

GOVERNING AUTHORITIES (NATIONAL AND LOCAL)

Topic 1: Introduction

Gender: ___ Male ____ Female

1. How long have you been working in South Sudan?
2. How long have you been working with [governing entity]?
3. What is your current position with [governing entity]? Could you tell me how long you have been working in this position?
4. Can you tell me about the roles and responsibilities of this position?

Topic 2: Coordination with other actors

1. In your position, have you participated in any cluster meetings? Within which clusters?
2. Can you describe what decisions are made at these meetings? Can you give an example?
3. Can you describe how [governing entity] coordinates health service delivery for women and children with UN or NGO agencies?

*Probe* (for national authorities only): How do you coordinate with local authorities?

*Probe* (for national authorities only): How does this vary across different parts of the country?

1. Can you describe any specific requirements that UN or NGO agencies must adhere to if they want to work within [South Sudan/specific site]?

*Probe*: Were visas or special permissions required for personnel entrance into the country or for the deployment of commodities, medications, or equipment? From whom were permissions sought?

*Probe*: Are any of these specific to health services for women and children?

Topic 3: Insecurity

1. We understand that working in South Sudan can be difficult given the ongoing insecurity. Can you describe the impact of the changing security context on planning and delivering health services for women and children?

*Probe*: To what extent were security considerations a basis for confining services to specific locations, such as IDP or refugee camp settings?

1. How did you manage the security challenges?

*Probe:* With whom did the providers of services negotiate security?

*Probe:* Did security forces accompany the transport of personnel, commodities, or equipment to service locations?

*Probe:* Were non-standard fees or covert payments required to operate facilities?

1. How has the impacts of the security situation changed over time?

*Probe*: How has it changed for humanitarian workers or facilities as victims of violence?

Topic 4: Prioritization

1. Just as a reminder we are focusing on the period between 2013 to 2017. Could you describe what informed or continues to inform your decisions on which interventions to deliver for reproductive, maternal, newborn, child, and adolescent health?
2. Can you describe what information is used to determine the health services provided for women and children in South Sudan?

*Probe*: What areas of health did these assessments focus on? What type of data was collected in these assessments? Can you describe the geographic areas where these assessments were conducted? How was the decision made to conduct assessments in these areas? Can you give me information about who conducted these assessments? Can we access these assessments?

1. Can you describe how the level of funding received by [governing entity] for work in South Sudan has affected what health interventions were prioritized?

*Probe:* Did the source of the funding influence the type of services delivered for women and children? Were there any restrictions associated with this funding? Were certain services deprioritized because of financial constraints? Can you give me an example?

1. Can you describe any other factors that influenced the prioritization of interventions for women and children?

Topic 5: Health workforce

1. Can you describe how the availability of certain cadres of health workers influenced priorities for reproductive, maternal, newborn, child, and adolescent health?
2. How do you manage health workforce turnover?
3. What challenges do you face with recruitment of health workers?
4. What areas within health are most affected by the availability of the health workers?

Topic 6: Essential commodities

1. Between 2013 and 2017, were there any concerns about the availability or shortages of commodities for women’s and children’s health? Can you describe any adjustments that were made to [specific intervention of interest] as a result of commodity availability?

Topic 7: Culture *(local level only)*

1. We would like to understand what influences the delivery of health interventions for women and children. Can you explain how cultural beliefs have influenced the acceptability of health interventions?

*Example of health interventions*: Family planning, antenatal care, care during childbirth, essential newborn care, postnatal care, PMTCT of HIV, applying nothing to the newborn’s cord, kangaroo mother care, immediate breastfeeding, care for sick children under five, child vaccination, management of acute malnutrition, care for survivors of sexual assault

*Probe*: Can you give me some examples?

1. Does culture affect service provision in one area of health in particular?

*Probe*: Can you give me an example?

1. Can you describe any subpopulations that were particularly hard to access?
2. Can you describe any special efforts to try to access these subpopulations?

Topic 8: Closing interview

1. Before we end, do you have any additional thoughts that were not shared? If you have any additional thoughts or questions, feel free to contact me.

That is the end of our interview today. Thank you so much for your time. Do you have any questions for me? *[Answer any questions.]*

**Interview Guide: In-Depth Interview with Donors**

IN-DEPTH INTERVIEW GUIDE

DONORS

Topic 1: Introduction

Gender: ___ Male ____ Female

1. How long have you been working in South Sudan?
2. How long have you been working with [agency]?
3. What is your current position with [agency]? Could you tell me how long you have been working in this position?
4. Can you tell me about the roles and responsibilities of this position?

Topic 2: Prioritization

1. Just as a reminder we are focusing on the period between 2013 to 2017. Could you describe what informed or continues to inform your decisions on which interventions to deliver for reproductive, maternal, newborn, child, and adolescent health?
2. Can you describe how needs assessments or surveys were utilized to determine the interventions [donor] provided in South Sudan?

*Probe*: What areas of health did these assessments focus on? What type of data was collected in these assessments? Can you describe the geographic areas where these assessments were conducted? How was the decision made to conduct assessments in these areas? Can you give me information about who conducted these assessments? Can we access these assessments?

1. Can you describe how scientific or academic literature influenced the interventions [donor] provided in South Sudan?
2. Can you describe how guidelines influenced the interventions [donor] provided in South Sudan?

*Probe:* Can you list which guidelines you or your organization uses?

1. Can you describe how the cost-effectiveness of different interventions influenced the interventions [donor] provided in South Sudan?
2. Can you describe how the level of funding received by [donor] for work in South Sudan has affected what health interventions were prioritized?

*Probe:* Did the source of the funding influence the type of services delivered? Were there any restrictions associated with this funding? Were certain services deprioritized because of financial constraints? Can you give me an example?

1. Can you describe any other factors that influenced the prioritization of interventions?

Topic 3: Coordination with government

1. Can you describe how [donor] coordinates funding of service delivery with government?
2. Can you describe any specific government requirements that [donor] must adhere to if they want to work within South Sudan?

Topic 4: Closing Interview

1. Before we end, do you have any additional thoughts that were not shared? If you have any additional thoughts or questions, feel free to contact me.

That is the end of our interview today. Thank you so much for your time. Do you have any questions for me? *[Answer any questions.]*

**Interview Guide: In-Depth Interview with Senior Technical Leads**

IN-DEPTH INTERVIEW GUIDE

SENIOR TECHNICAL LEADS (NATIONAL AND LOCAL)

Topic 1: Introduction

Gender: ___ Male ____ Female

1. How long have you been working in South Sudan?
2. How long have you been working with [agency]?
3. What is your current position with [agency]? Could you tell me how long you have been working in this position?
4. Can you tell me about the roles and responsibilities of this position?

*Probe* (only if generalist): Can you describe specific responsibilities for providing health services for women and children?

Topic 2: Scope of work

1. We have been looking at [agency] activities in South Sudan and have made a list of the services and programs that [agency] delivers for reproductive, maternal, newborn, child and adolescent health. Can you let me know if anything is missing from the list or anything that is no longer delivered? (*share list with participant)*

Topic 3: Organizational expertise

1. Does your team have expertise in one area of health? How has that influenced prioritization of health interventions?
2. Can you describe how you manage competing priorities?

*Probe:* In what sense were these priorities competing? Time? Workforce? Resources?

Topic 4: Coordination with other actors

1. In your position, have you participated in any cluster meetings? Within which clusters?
2. Can you describe what decisions are made at these meetings? Can you give an example?
3. Can you describe how [agency] coordinates funding of service delivery for women and children with [local/national] authorities?
4. Can you describe any specific [local/national] requirements that [agency] must adhere to if they want to work within South Sudan?

*Probe:* Special permissions for personnel entrance into the country? Selecting the location for service delivery?

Topic 5: Insecurity

1. We understand that working in South Sudan can be difficult given the ongoing insecurity. Can you describe the impact of the changing security context on service delivery for women and children?

*Probe*: Can you give me some examples?

1. How did you manage the security challenges?
2. How has the impact of the security situation changed over time?

Topic 6: Prioritization

1. Just as a reminder we are focusing on the period between 2013 to 2017. Could you describe what informed or continues to inform your decisions on which interventions to deliver for reproductive, maternal, newborn, child, and adolescent health?
2. Can you describe how needs assessments or surveys were utilized to determine the interventions [agency] provided in South Sudan?

*Probe*: What areas of health did these assessments focus on? What type of data was collected in these assessments? Can you describe the geographic areas where these assessments were conducted? How was the decision made to conduct assessments in these areas? Can you give me information about who conducted these assessments? Can we access these assessments?

1. Can you describe how scientific or academic literature influenced the interventions [agency] provided in South Sudan?
2. Can you describe how global, national or agency-specific guidelines influenced the [agency] provided interventions for women and children in South Sudan?

*Probe:* Can you list which guidelines you or your organization uses for reproductive, maternal/newborn, child and adolescent health, or nutrition?

1. Can you describe how the cost or cost-effectiveness of different interventions influenced the interventions [agency] provided in South Sudan?
2. (*national level only*) Can you describe how the level of funding received by [agency] for work in South Sudan has affected what health interventions were prioritized?

*Probe:* Did the source of the funding influence the type of services delivered? Were there any restrictions associated with this funding? Were certain services deprioritized because of financial constraints? Can you give me an example?

1. Can you describe any other factors that influenced the prioritization of interventions?

Topic 7: Population displacement

1. Were there any large influxes of displaced populations? Can you describe how this influx influenced your service provision?

*Probe*: Can you give me some examples?

1. Did you receive additional support to provide services to the influx of population?

Topic 8: Health workforce (*local level only)*

1. Can you describe how the availability of certain cadres of health workers influenced [specific intervention of interest] priorities?
2. How do you manage health workforce turnover?
3. What challenges do you face with recruitment of health workers?
4. What areas within health are most affected by the availability of the health workers?

Topic 9: Essential commodities (*local level only)*

1. Between 2013 and 2017, were there any concerns about the availability or shortages of commodities for women’s and children’s health? Can you describe any adjustments that were made to [specific intervention of interest] as a result of commodity availability?

Topic 10: Epidemics (*local level only)*

1. Were there any epidemics while you were working here? Can you describe how this influenced RMNCAH/N service provision?

*Probe*: Can you give me some examples?

1. Did you receive additional support to address the epidemic?

Topic 11: Closing interview

1. Before we end, do you have any additional thoughts on service delivery strategies and challenges for women and children that were not shared? If you have any additional thoughts or questions, feel free to contact me.

That is the end of our interview today. Thank you so much for your time. Do you have any questions for me? *[Answer any questions.]*
